# Oxford Handbook of Oncology

**DOI:** 10.1038/sj.bjc.6603701

**Published:** 2007-04-17

**Authors:** W P Steward

**Affiliations:** 1 Leicester Royal Infirmary, Leicester, UK

The first edition of the Oxford Handbook of Oncology appeared in 2002 and was designed to provide essential information points – often as bulleted highlights – on the background of Oncology, general therapeutic options, Prevention, and subsequent chapters relating to specific tumour types. There were additional brief sections on Oncological Emergencies and new developments in therapy. As such, it provided a useful rapid reference text, particularly aimed at students and trainees but also providing quick reference guides for experienced Oncologists, particularly when looking for information on areas outside their specific expertise. Although the first edition had many commendable features, there were significant drawbacks in terms of inconsistencies between chapters particularly relating to the breadth of information provided for different tumour types, varying styles and frequent omission of new therapies – particularly in Sections relating to breast cancer and lymphomas.

It is encouraging that, in this second edition, the authors have addressed the deficiencies of its predecessor while retaining the important overall aim of producing a concise and comprehensive cover of the most important aspects of Oncology. For each tumour type, there is now a common approach to layout to include pathology, staging, modern imaging and therapy.

The book is now divided into eight parts, the first including brief but comprehensive overviews of the aetiology and epidemiology of cancer, together with a very brief overview of the genetics of malignancy. This is followed by chapters relating to different aspects of the principles of treatment including an excellent overview of radiation oncology, which is particularly helpful for a non-specialist. There is a comprehensive summary of the principles of chemotherapy that outlines the use of the most important anticancer drugs and contains key information with lists of important toxicities. There is particular emphasis on mechanisms of action and resistance. This section should be particularly useful as a rapid reference for trainees.

The subsequent section relating to principles of prevention and population screening is brief but contains valuable information at an appropriate level for the target audience. The following sections relating to major tumour types are uniformly well written and laid out in a logical sequence containing details of aetiology, epidemiology, pathology, staging and management. Information is predominantly included as key bullet points which are easy to rapidly scan when seeking particular items. Most are fully updated with modern therapeutic approaches, although some are, inevitably, slightly out-of-date with, for example, no mention of the potential value of angiogenesis inhibitors in renal cancer and considerable emphasis on different approaches to administration of fluorouracil (5FU) and folinic acid for colorectal cancer and less information on more modern approaches with combination regimes and targeted therapies. The final sections covering symptom control and emergencies in oncology are particularly well written and will be of great value for all levels of readership. Chapters on palliative care have been extensively revised and expanded by 50% to provide an excellent overview of symptom control – in particular pain management – and to cover psychological problems related to cancer.

In summary, the Oxford Handbook of Oncology retains all the positive features of its predecessor, being concise and containing key pieces of information on a huge variety of topics relating to the understanding of carcinogenesis from prevention to management of different malignancies. It represents a considerable improvement on its predecessor having undergone expansion of some of the key sections, a more uniform style in each of the descriptions of tumour types and significant updating of information on therapeutic approaches. It should provide a valuable rapid reference for a wide variety of clinicians, oncologists in training, nurses and students.

